# A mysterious foe in a case of pancytopenia with splenomegaly: *Plasmodium vivax* gametocytes in the bone marrow

**DOI:** 10.1016/j.htct.2026.106354

**Published:** 2026-03-21

**Authors:** Govind R. Patel, Vikram Singh

**Affiliations:** Department of Clinical Hematology, Dr. SN Medical College, Jodhpur 342003, Rajasthan, India

## Introduction

*Plasmodium vivax*, the most widely distributed human malaria parasite, poses a significant disease burden [1]. *P. vivax* usually causes a less severe form of malaria compared to *Plasmodium falciparum*, as it is rarely associated with severe complications but it can cause severe anemia and severe thrombocytopenia [1,2]. Pancytopenia with splenomegaly as an initial manifestation of acute *P. vivax* malaria is extremely uncommon; it is mainly reported after chronic or repeated exposure to *P. vivax*, as a manifestation of hypersplenism associated with hyperactive malarial splenomegaly syndrome. Pancytopenia with splenomegaly as a primary presentation is more often associated with *P. falciparum*.

Diagnosis of *P. vivax* malaria typically involves a peripheral blood smear (PBS) to detect the parasite. Even though *P. vivax* can be found in the bone marrow, its presence in this tissue without being present in the peripheral blood has rarely been documented [3]. To the best of our knowledge, there are no documented cases of pancytopenia with splenomegaly associated with acute *P. vivax* malaria with the presence of its gametocytes only in the bone marrow and not in PBSs.

Recognizing the unusual presentation of *P. vivax* malaria is essential for early diagnosis and management. We report a rare case of acute *P. vivax* malaria presenting with fever, pancytopenia and splenomegaly which incidentally showed gametocytes of *P. vivax* in a bone marrow smear without any parasite stage in PBS. The patient had a full recovery from fever, pancytopenia and splenomegaly with oral chloroquine and primaquine. We believe that physicians from endemic countries for malaria such as India should be aware of such a presentation.

## Case report

**Figure 1:**
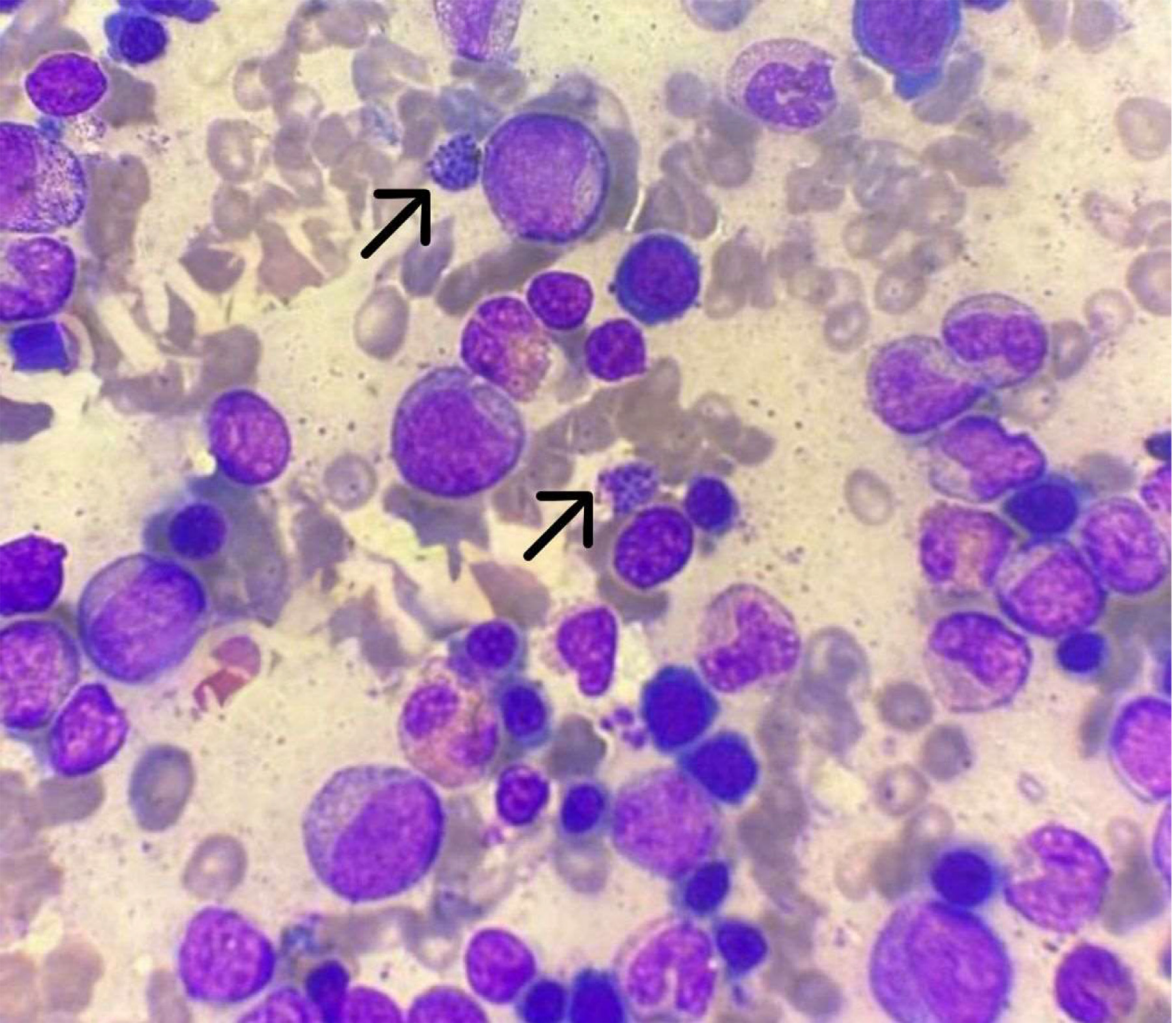
Presence of *Plasmodium vivax* gametocytes in a bone marrow aspirate.

A 47-year-old woman from western India, a malaria endemic region, with unremarkable medical history and in overall good health initially visited her local hospital for primary care due to a high-grade fever of one week duration. Routine laboratory investigations were performed, including a rapid diagnostic test (RDT) and microscopic examination of PBS; both were negative for malaria parasites, and no hematological abnormalities were detected. Symptomatic medications and antibiotics were given, but the fever persisted for more than three weeks. During this period, she developed easy fatigability, and clinical examination revealed a palpable spleen. Subsequent laboratory evaluations showed persistent pancytopenia despite serial RDTs and PBS remaining negative for malaria. Due to the suspicion of a hematological malignancy, the patient was referred to our center for further diagnostic evaluation. Upon presentation, she was febrile with a temperature of 39 °C (102.3°F). She was very pale and had a palpable splenomegaly (7 cm below left costal margin) on the physical examination. The rest of the physical examination was unremarkable. Her initial laboratory findings revealed pancytopenia with hemoglobin (Hb) of 5.3 g/dL, total leukocyte count of 2.4 × 10^3^/µL with absolute neutrophil count of 0.8 × 10^3^/µL, and a platelet count of 27 × 10^3^/µL. She had a reticulocyte count of 4.5 % with a corrected reticulocyte count of 1.9 %. The PBS showed predominantly microcytic hypochromic erythrocytes, leucopenia (neutropenia and lymphopenia), and reduced platelets on smear, without any evidence of ring forms, trophozoites or schizonts of *P. vivax*. Biochemical tests revealed normal levels of serum ferritin (131.7 ng/mL), vitamin B12 (490 pg/mL) and triglyceride (110 mg/dL) with a mildly raised serum lactate dehydrogenase level (227.15 U/L). Her plasma fibrinogen level was normal (420 mg/dL) and glucose-6-phosphate dehydrogenase activity was normal (32.31 U/g Hb). A direct Coombs test was negative. Renal and liver function tests were normal. Serologic tests for hepatitis B and C virus, human immunodeficiency virus, and cytomegalovirus were negative. Blood and urine cultures showed no growth. Her abdominal ultrasound confirmed a moderate splenomegaly (spleen size 17.8 cm). Since there were signs and symptoms mimicking a hematological malignancy, a bone marrow examination (aspirate and biopsy) was performed which surprisingly revealed *P. vivax* gametocytes in the bone marrow smear with mild erythroid hyperplasia and dyserythropoiesis, normal myelopoiesis, and an adequate number of functioning megakaryocytes (Figure 1). Based on the clinical, hematological and biochemical parameters, and bone marrow findings, a final diagnosis of pancytopenia with splenomegaly as a result of *P. vivax* infection was established. This is a rare case because acute *P. vivax* malaria presented with pancytopenia and moderate splenomegaly along with the presence of *P. vivax* gametocytes in the bone marrow without any evidence of the parasite in the initial work-up, particularly in the PBS. Antimalarial treatment was administered following the standard guidelines (1500 mg of oral chloroquine over three days and 15 mg of primaquine per day for 14 days: the primaquine was administered for radical treatment of *P. vivax*) along with iron supplements. Her condition improved rapidly after starting the treatment with fever subsiding after three days of chloroquine. During her follow-up visits, her hematologic parameters had normalized. The pancytopenia and splenomegaly were fully resolved by the 28th day after discharge. At six weeks after treatment, repeat bone marrow aspirate and biopsy samples were obtained for comparison with the initial findings. A microscopic bone marrow smear examination was negative for gametocytes. Hematological parameters at baseline and follow-ups are shown in Table 1.

**Table 1:** Hematological parameters at baseline and at two and six weeks of follow-up.

Parameter	Baseline	2 weeks	6 weeks
Hemoglobin (g/dL)	5.3	8.0	12.5
Hematocrit ( %)	18.6	24.6	41.3
Total erythrocyte count (10^6^/µL)	3.36	3.98	4.65
Mean corpuscular volume (fL)	55.4	61.8	81.9
Mean corpuscular hemoglobin (pg)	15.8	20.1	26.9
Mean corpuscular hemoglobin concentration (g/dL)	28.5	32.5	32.8
Total leukocyte count (10^3^/µL)	2.40	3.92	8.93
Absolute neutrophil count (10^3^/µL)	0.80	1.86	6.30
Platelet count (10^3^/µL)	27	80	371
Reticulocyte count ( %)	4.5	4.2	1.3
Corrected reticulocyte count ( %)	1.94	2.30	1.19
Reticulocyte production index	0.8	1.1	1.2

## Discussion

*P. vivax* malaria is a significant global health issue in many countries, particularly in tropical and subtropical regions, including sub-Saharan Africa, Asia, and Latin America [1,4]. Contrary to the general perception that *P. vivax* malaria usually results in minimal complications, a series of reports have demonstrated that the infection can cause multiple-organ dysfunction and severe life-threatening complications such as severe anemia, hepatic dysfunction and jaundice, acute lung injury, acute respiratory distress syndrome and pulmonary edema, shock, acute kidney injury, severe thrombocytopenia and splenic rupture [1,2].

This case presented with a prolonged fever, splenomegaly, pancytopenia, and easy fatigability, the signs and symptoms mimicking a hematological malignancy. *P. vivax* was not identified in the preliminary work-up (RDT and PBS examinations); quantitative polymerase chain reaction is not a routine work-up for Plasmodium suspected patients in our country. The patient underwent extensive work-up to exclude other infectious diseases. Our patient incidentally demonstrated *P. vivax* gametocytes in the bone marrow smear, indicating that the parasites can invade the bone marrow, leading to pancytopenia and other hematological abnormalities.

Pancytopenia due to *P. vivax* malaria is extremely rare and so far has been documented in 0.9 % of confirmed *P. vivax* cases [4]. Pancytopenia as a primary presentation is uncommon in *P. vivax* malaria; it is more often associated with *P. falciparum. P. vivax* malaria may cause pancytopenia through several mechanisms, including hemophagocytic lymphohistiocytosis, myelosuppression, hypersplenism, or tumor lysis by infection-related steroid release [4]. However, the underlying pathogenic mechanisms of pancytopenia remain largely unresolved in our case as all these mechanisms are unlikely. Further research is needed to understand the infection-related reduced blood cell counts.

The spleen plays a crucial role in filtering the blood and removing infected red blood cells, leading to its enlargement during malaria infection. Splenomegaly, one of the most common features of malaria, is directly related with severity [5]. Spleen enlargement occurs if an individual experiences parasitemia for a period exceeding two weeks. Thereafter the degree of spleen enlargement depends upon the duration of exposure and severity of parasitemia [5]. Splenomegaly during acute malaria infections is more common with *P. falciparum* than with *P. vivax*, as the former is more frequently associated with high parasite densities, leading to increased clearance of both parasitized and non-parasitized erythrocytes [5]. Soni & Jalaly observed splenomegaly in 41 % of *P. falciparum* and 20 % of *P. vivax* patients [5]. Strickland et al. observed that greater splenic volume was positively correlated with the likelihood of *P. falciparum* infection, whereas *P. vivax* was more frequently associated with mild splenomegaly [6]. Splenomegaly in acute *P. vivax* malaria can be associated with a higher risk of spontaneous splenic rupture, a serious and potentially life-threatening complication [1]. The spleen could be a niche for *P. vivax* leading to splenomegaly and maintenance of a low peripheral parasitemia [1].

In the present case, repeated thin and thick PBSs and RDTs failed to demonstrate *P. vivax*. Because the clinical presentation was suggestive of a hematological malignancy, a bone marrow examination was performed as part of the diagnostic evaluation. *P. vivax* parasites can be found in the bone marrow during an active infection. The presence of *P. vivax* in the bone marrow was first noticed in the late 19th century [3,7]. Sternal bone marrow aspirate examinations used to be performed as an accessory to peripheral blood examination in malaria [8]. Baro et al. showed that *P. vivax* gametocyte stage-infected cells are enriched in the bone marrow compared to peripheral blood during the acute infection [3]. These data suggest that the bone marrow could also be a reservoir for gametocytes during *P. vivax* infections. Whether the bone marrow functions as a niche for gametocyte production or maturation, and whether these forms sequester there as observed in *P. falciparum*, warrants further investigation in future studies. Our case suggests that bone marrow aspiration is of value in the diagnosis of malaria.

In the present case study, the repeated RDTs for the malarial antigen were negative, and the *P. vivax* was also not detected in repeated PBS examinations. RDTs for *P. vivax* have relatively poor performance compared with those for *P. falciparum* as uptake is slow and inconsistent. This is due to a combination of lower parasite density in *P. vivax* infections, lower expression of the parasite lactate dehydrogenase, and poorer performance of the reagents used for this specific antigen. As a consequence, many RDTs might fail to detect *P. vivax* in samples containing ≤200 parasites/µL [9]. The negative results of serial RDTs in this case was possibly due to false-negative results. On the other hand, the negative PBS for *P. vivax* may be attributed to the pretreatment with antimalarial drugs in inadequate doses, causing partial clearance of the parasite, low levels of parasitemia not detected by conventional microscopy or by sequestration of the parasitized cells, in deep vascular beds [4].

## Conclusion

*P. vivax* malaria should be considered in the differential diagnosis in all people from endemic areas who present with fever, pancytopenia and splenomegaly. A diagnostic bone marrow examination should be recommended in patients from endemic regions with negative results for *P. vivax* in serial RDTs and microscopic PBS examinations after all other possible infectious causes are excluded, since early diagnosis and treatment of malaria are crucial to prevent serious complications and mortality.

## Authors’ contributions

Both authors meet the ICMJE authorship criteria. GRP designed the study, collected data, and contributed to writing, reviewing and editing the manuscript with overall supervision. VS collected raw patient data, obtained the patient consent and wrote the first draft of the manuscript. Both authors read and approved the final manuscript.

## Ethics approval

Ethical approval for this case report was given by the Institutional Ethics Committee.

## Consent for publication

The patient provided written informed consent for the publication of this case report.

## Availability of data and materials

The authors confirm that the data generated and analyzed in this study are included in this published article.

## Funding

The authors did not receive any financial support for the purpose of this case report.

## Conflicts of interest

The authors declare no competing financial or other conflicts of interest.
